# A Tale of Two RNAs during Viral Infection: How Viruses Antagonize mRNAs and Small Non-Coding RNAs in The Host Cell

**DOI:** 10.3390/v8060154

**Published:** 2016-06-02

**Authors:** Kristina M. Herbert, Anita Nag

**Affiliations:** 1 Department of Experimental Microbiology, Center for Scientific Research and Higher Education of Ensenada (CICESE), Ensenada, Baja California 22860, Mexico; 2 Department of Chemistry, Florida A&M University, Tallahassee, FL 32307, USA

**Keywords:** mRNA, endonuclease, host shut-off, virus, RNAi

## Abstract

Viral infection initiates an array of changes in host gene expression. Many viruses dampen host protein expression and attempt to evade the host anti-viral defense machinery. Host gene expression is suppressed at several stages of host messenger RNA (mRNA) formation including selective degradation of translationally competent messenger RNAs. Besides mRNAs, host cells also express a variety of noncoding RNAs, including small RNAs, that may also be subject to inhibition upon viral infection. In this review we focused on different ways viruses antagonize coding and noncoding RNAs in the host cell to its advantage.

## 1. Introduction

Viral infection has significant effects on the host’s gene expression program. Upon viral infection, mammalian cells activate specific gene expression pathways as a defense response. To assure survival, viruses manipulate the host’s gene expression response pattern in order to maximize viral replication and/or to evade detection by, and block the, host immune responses. Gene expression studies have typically focused on protein-coding messenger RNAs (mRNAs). However, many viruses also either significantly affect the expression of genomic encoded mammalian non-coding RNAs, such as small RNAs, or encode their own small RNAs. These small RNAs might either down-regulate levels of the proteins encoded by target mRNAs, thus having significant effects on the host cell phenotype or actually target the viral genome for degradation. In this review, we focus on the RNA-based ways in which mammalian viruses (Table 1) antagonize host gene expression pathways to their advantage. We first touch upon several pathways that exist to shutdown host mRNAs by mammalian viruses, either transcriptionally or post-transcriptionally. We highlight the recent and substantial mechanistic studies looking at how viruses induce the degradation of host mRNAs in order to maximize the expression of their own genes and their rate of replication. In the second part of the review, we discuss how many viruses also antagonize host small noncoding RNAs. Since we are focused on mammalian viruses, we examine the controversy surrounding whether the anti-viral RNA interference (RNAi) based response in lower eukaryotes also exists in mammals. Finally, in the last section, we review how some viruses have taken advantage of the miRNA pathway and degrade or inhibit microRNAs (miRNAs) in order to maximize viral replication and manipulate cell-signaling pathways. Viruses that are highlighted in this review are presented in Table 1.

## 2. Shutting down Cellular mRNA

Upon viral infection, viruses often down-regulate host gene expression in order to maximize the expression of their own genes or in order to maximize their chances of replicating. Down-regulation of host gene expression can take place either co-transcriptionally in the nucleus or post-transcriptionally in the nucleus or cytoplasm. Inactivation of transcription factors to prevent their assembly on RNA polymerase II (RNAPII) driven promoters is often a mechanism that stops transcription at an early stage. Inactivation of RNAPII driven transcription of host mRNAs can be global, but in certain viruses, for example in Rift valley fever virus (RVFV), transcription from selected genes is blocked [1]. Moreover, some viruses have developed ways to block multiple steps leading to the formation of a mature mRNA including transcription elongation, processing, and export [2]. Another important mechanism to down-regulate host gene expression is by destabilization of the mRNA. This can be achieved by decapping or endonucleolytic cleavage of the mRNA followed by exonucleolytic decay. Several DNA and RNA viruses target mRNAs that are undergoing ribosome assembly, and cleave the mRNA for subsequent degradation [3]. In the first section of the review, we will only briefly discuss representative viruses that use the first two categories of mRNA suppression during transcription as these topics are reviewed broadly elsewhere [4]. We will elaborate more on the mechanism of mRNA down-regulation by degradation through cleavage, as this mechanism has been more recently demonstrated to be an essential host shut-off pathway for several DNA and RNA viruses, mostly in herpesviruses and coronaviruses. Although these are two different families of viruses, their host shut off through mRNA cleavage mechanisms are strikingly similar. The herpesvirus family consists of DNA viruses that have both latent and lytic phases of infection, with host-shut off only starting upon lytic reactivation. While members of the coronavirus family show a similar host mRNA cleavage-degradation mechanism, in these positive strand RNA viruses host shut-off starts immediately after viral proteins are available.

### 2.1. Transcriptional Down-regulation of mRNAs by Viral Proteins

**Figure 1:**
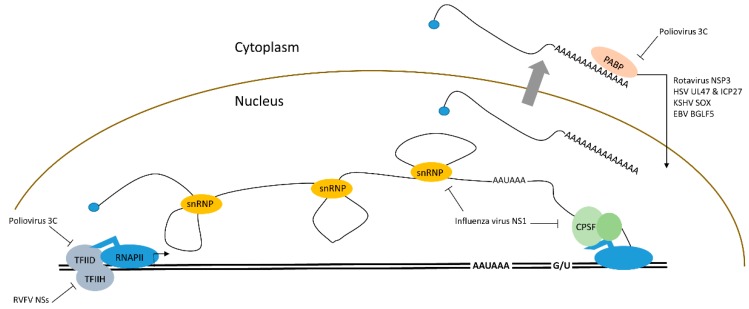
Inhibition of host pre-mRNA transcription and processing by specific viral proteins. Poliovirus protein 3C and RVFV protein NSs block the initiation of RNA polymerase II (RNAPII) at promoter sequences by inactivating transcription factor II H (TFIIH) or transcription factor II D (TFIID), respectively. The influenza virus protein NS1 blocks pre-mRNA cleavage by inhibiting cleavage polyadenylation factor CPSF and poly(A) binding protein PABP. NS1 also blocks pre-mRNA splicing by interfering with the small nuclear ribonucleoprotein (snRNP) complex. In addition, localization of poly(A) binding protein PABP is manipulated by several of the indicated viruses to dampen RNA stability, transport, and mRNA translation.

Suppression of host mRNA transcription is a natural choice for many viruses that down-regulate gene expression. Viral proteins block RNA transcription and processing through their interaction with cellular proteins (Figure 1). In the picornavirus family, poliovirus encodes the 3C protease that inactivates the first basal transcription factor that initiates mRNA transcription from RNAPII driven promoters, transcription factor II D (TFIID). Protease 3C proteolytically cleaves one of the three Gln-Gly bonds in TFIID [5,6]. Similarly, the vesicular somatic virus (VSV) encoded noncatalytic matrix (M) protein also inhibits TFIID [7]. RVFV is a bunyavirus that has high fatality in sheep and cattle, but also affects humans, causing fatal hemorrhagic fever. The nonstructural proteins (NSs) of RVFV localize in the nucleus and interact with transcription factors including transcription factor II H (TFIIH) [8]. A recent work by Benferhat *et al.* found NSs interact with the promoter region of selective genes including genes that regulate innate immunity, inflammation, cell adhesion, and even promoters of coagulation factors [1]. Old world alphaviruses (Sindbis virus, Chikungunya virus) induce degradation of the RNAPII large subunit (Rpb1) by viral protein NSP2 mediated ubiquitination of Rpb1 [9]. After transcription initiation, pre-mRNA processing factors may also be inhibited by viral proteins. Influenza virus nonstructural protein 1 (NS1) can shut down host mRNAs by several mechanisms. First, it binds to the 30 kDa subunit of the cleavage polyadenylation specificity factor (CPSF) [10]. Both transcription termination and RNA processing are disrupted by this interaction [11]. NS1 also inhibits poly(A) binding protein (PABP) causing disruption of polyadenylation, which inhibits mRNA export to the cytoplasm [12]. Finally, NS1 also interferes with binding of U6 RNA to U2 and U4 during spicing, thus NS1 inhibits RNA cleavage, polyadenylation, and splicing [13]. While interaction with transcription initiation and processing factors can inhibit proper host mRNA production, examples exist in herpesviruses where viral factors bind to either TFIID (herpes simplex virus 1 (HSV-1) protein ICP4) or specifically the TFIID subunit 4, TAF4, (Epstein Barr virus (EBV) protein Rta) to selectively induce RNAPII transcription of early viral transcripts from viral promoters [14,15]. This mechanism ensures upregulation of viral transcripts over cellular transcripts.

### 2.2. Post-Transcriptional Modification: Decay of mRNA by Decapping

**Figure 2:**
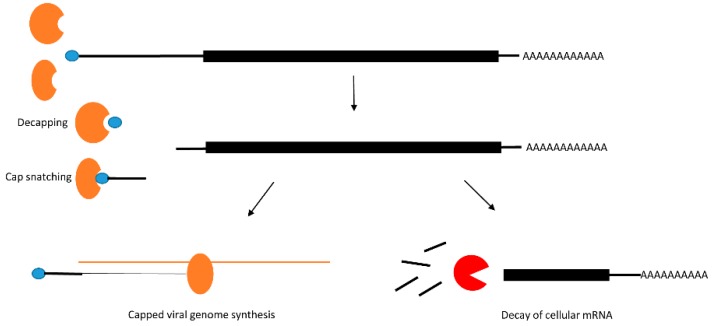
Decapping, cap snatching and cellular mRNA decay. In the case of VACV, the 5′ protective cap of the mRNA is removed by the viral decapping proteins (D9 or D10), which then allows for the host protein XRN1 to degrade the RNA. In Influenza virus, cap snatching by the PA protein initiates viral RNA synthesis that uses the removed cap as a primer leaving uncapped cellular mRNAs for degradation.

In uninfected cells mRNAs are protected by the virtue of possessing 5′-caps and 3′-poly(A) tails that protect the RNA from exonucleases that remove nucleotides from the 5′-end (XRN1 primarily in the cytoplasm and XRN2 in the nucleus) or the 3′-end (the exosome complex). Poxviruses use virally encoded decapping proteins to remove the 5′-cap of the mRNA resulting in destabilization of the mRNA. Other viruses including bunyaviruses and orthomyxoviruses use cap-snatching mechanisms to not only remove the 5′-cap, but then use the removed cap for protecting viral RNAs (Figure 2). This mechanism and various viral examples are well described in the review by Narayanan and Makino [16]. In bunyaviruses (negative stranded RNA viruses. e.g., RVFV), the virus encoded nucleocapsid (N) recognizes the 5′-cap of the mRNA and a 10–18 nucleotide region, while the viral RNA dependent RNA polymerase (RdRP) L, cleaves the RNA and uses the capped RNA fragment as a primer to synthesize capped viral mRNA. Interestingly these proteins localize in the processing bodies (P-bodies) where they compete with the cellular decapping enzyme Dcp2 for cell cycle regulated mRNAs [17]. The 5′-cap is recognized by the influenza virus PB2 subunit of the viral RNA polymerase in the nucleus of the host cell, while the endonucleolytic function is carried out by the polymerase subunit PA (polymerase acidic protein) [18]. However, in bunyaviruses and arenaviruses, cap-snatching happens in the cytoplasm. Poxviruses, a class of viruses with dsDNA genomes that are uniquely replicated exclusively in the cytoplasm, all express their own mRNA decapping enzymes. The prototypical poxvirus, Vaccinia virus (VACV) expresses two decapping enzymes, D9, which has a homolog in nearly all vertebrate poxviruses, and D10, which has conserved homologs in all poxviruses. Upon VACV infection, D9 and D10 are expressed during the early and late stages of infection and are suggested to target host mRNAs so that the available translation machinery can be used for viral RNA translation only [19,20].

### 2.3. Translational Inhibition with and without mRNA Cleavage

**Figure 3:**
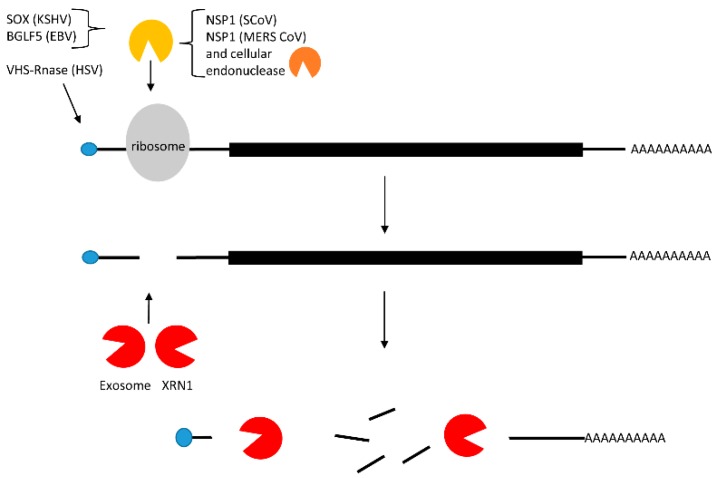
Endonucleolytic cleavage of mRNA in the 5′ UTR. Viral proteins SOX (Kaposi sarcoma herpes virus, KSHV), BGLF5 (Epstein Barr virus; EBV), VHS RNase (herpes simplex virus 1; HSV-1), and NSP1 (severe acute respiratory syndrome corona virus; SCoV) bind to the ribosome on translating mRNAs. RNA is cleaved in the 5′-UTR by viral or cellular endonucleases. Cleaved mRNAs are degraded by the exosome in the 3′→5′ direction, and by XRN1 5′→3′.

Both cellular and viral mRNAs use host ribosomes for translation. Adventitiously, many viruses inhibit host mRNA translation to fully exploit cellular translation factors to translate their own viral mRNAs. Host translational inhibition can be indirectly achieved by dampening transcription and RNA processing (as discussed above). However, many eukaryotic viruses, including both DNA and RNA viruses, trigger mRNA degradation either by a virally encoded endonuclease, or in some cases they make use of a host endonuclease to cleave the cellular mRNA [21]. In this section of the review we discuss several examples of viral factors that show a high level of similarity in their mechanism of cleavage and degradation of host mRNAs (Figure 3). We compare and contrast the host shut-off pathways of the DNA viruses, Kaposi sarcoma herpes virus (KSHV) and EBV), which are both gammaherpesviruses, the herpes simplex virus 1 (HSV-1), which is an alphaherpesvirus, as well as the RNA virus, severe acute respiratory syndrome corona virus (SARS CoV).

In the latent phase of infection by KSHV and EBV, the majority of viral gene expression is restricted—perhaps to avoid immune system surveillance. During the lytic phase, viral gene expression is upregulated with a concomitant down-regulation of cellular mRNAs through host shut-off mechanisms. This shut-off of host mRNAs is initiated during the early lytic phase when the viral KSHV SOX (shut-off and exonuclease) or its EBV homolog BGLF5 are first expressed. SOX and BGLF5, are both alkaline exonucleases that induce DNA exonucleolytic cleavage, which is required for the processing and encapsidation of the viral genomic DNA. However, in addition, they also induce host mRNA degradation initiated by endonucleolytic cleavage. When expressed transiently or during the lytic phase of the viral life cycle, SOX is found in both the nucleus and in the cytoplasm [22,23]. Interestingly, the function of the protein is distinct in different compartments. In the nucleus, SOX is responsible for its exonucleolytic DNase activity, but its host shut-off function is confined to only the cytoplasm [23]. Similar to SOX, BGLF5 contains two nuclear localization signals (NLS), and is also present in both the nucleus and cytoplasm [24].

Among alphaherpesviruses, HSV-1 encodes a virion host shut-off (VHS) RNA endonuclease protein that selectively diminishes stable host gene expression as well as viral immediate-early gene expression by interfering with mRNA translation [25]. VHS-RNase contains a nuclear export signal (NES), but no NLS. Upon infection VHS-RNase gets localized to the nucleus, then shuttles back to the cytoplasm where it specifically disrupts cellular mRNA stability through its binding to the translation initiation factor, eIF4H [26]. 

A similar host shut off pathway has been discovered for coronaviruses. The β-coronaviruses, SARS CoV and Middle East respiratory syndrome corona virus (MERS CoV), initiate host shut-off using nonstructural protein NSP1 [27,28,29,30,31]. Surprisingly, NSP1 has no known endonuclease activity, but it is rather suspected to function by recruiting a cellular nuclease. Both SARS CoV NSP1 and its homolog encoded by MERS CoV share significant sequence similarities, but the host shut-off upon MERS CoV infection seems to be initiated in the nucleus while upon SCoV infection host shut-off is confined to the cytoplasm [31]. Unlike the β-coronaviruses that carry out host shut off by cleavage and degradation of mRNAs, the α-coronaviruses that include HCoV-229E and transmissible gastroenteritis virus dampen host gene expression without RNA cleavage. A thorough comparison of α- and β-coronaviruses is presented in a recent review [32].

Several aspects of host shut-off mechanisms are common among SOX, BGLF5, murine SOX (murine gamma herpesvirus 68), HSV-1 VHS, and SARS CoV NSP1. Most pathways target Pol II transcripts even if the target is not selected through the 5′ cap or poly(A) tail [3]. VHS interacts with translation initiation factors eIF4H and eIF4A, while NSP1 interacts with the 40S ribosome. Both SOX and NSP1 stall translation after the 40S ribosome complex has bound mRNA, blocking formation of the 80S complex [33]. Table 2 summarizes several mRNA suppression strategies discussed here.

Beyond this common endonuclease-based strategy, some viruses possess complex multi-step approaches to block gene expression of the host. In addition to degrading the host mRNA through nucleolytic cleavage, both SOX and BGLF5 participate in relocating proteins required for the stabilization of Pol II polyadenylated RNAs, such as poly(A) binding protein C (PABPC), from the cytoplasm to the nucleus. This relocalization ensures the instability of host mRNAs [34,35,36]. RVFV encoded NSs also induces relocalization of poly(A) binding protein 1 (PABP1) to the nucleus where it forms nuclear speckles. Since PABP1 interacts with several translation factors, relocalization of PABP1 to the nucleus is known to be responsible for inhibition of protein translation. Rotavirus and HSV-1 infection also induce the nuclear relocalization of PABP1, while during poliovirus infection PABP1 is targeted by the 3C protease. A similar but opposite relocalization of human antigen R (HuR) protein from the nucleus to cytoplasm is induced in alphaviruses (e.g., SINV) of family *Togaviridae* and the viral mRNA 3′UTR participates in capturing HuR in the cytoplasm, thereby selectively destabilizing a set of host mRNAs and their pre-mRNA processing that depends on HuR binding [37].

Upon KSHV infection, nuclear mRNAs get hyperadenylated in the presence of SOX protein [34]. Investigations into the mechanism that promotes hyperadenylation in the nucleus and the presence of any factors that mark these RNAs for degradation is yet to be investigated. Perhaps the nuclear polyadenylation and decay complex containing Trf4 homologs are responsible for unregulated adenylation of mRNAs which then targets them for decay [38]. Moreover, during the lytic phase KSHV encodes polyadenylated and nuclear (PAN) RNA that is both intronless and noncoding but is capped and polyadenylated. The PAN RNA possesses a U-rich expression and nuclear retention element (ENE) at its 3′-end [36,39,40] that along with the nearby poly(A) tail fold to form a triple helix structure, thereby protecting PAN RNA from the degradation that cellular RNAs are subjected to [41]. By overpopulating the nucleus with PAN RNA the virus ensures that PABPC is captured and sequestered in the nucleus [3,33,42]. Similar to KSHV, EBV also presents a similar mechanism in which PABPC is relocalized into the nucleus upon EBV infection. While BGLF5 alone can relocate PABPC into the nucleus where it accumulates in aggregates, another viral protein, ZEBRA, is also able to direct the intracellular relocalization of PABPC [35]. Unlike KSHV, there is no report of a PAN-like RNA in EBV to support a similar sequestration of PABPC in the nucleus. 

The HSV-1 host shut-off pathway is a complex process that must discriminate the normally stable host mRNAs from host stress response mRNAs. VHS-RNase binds to the cap structure of stable host mRNAs via its interaction with eIF4H. HSV-1 RNase acts differently on stress response RNAs that carry AU-rich elements (ARE) in their 3′-ends. These RNAs undergo cleavage in their 3′ UTRs, but the capped 5′-end of the RNA is stabilized and accumulates in P bodies. The KSHV-encoded protein SOX targets the 5′-UTR of most cellular mRNAs with some exceptions. One report suggests a concentration dependent down-regulation of mRNAs [43]. Among mRNAs that are resistant to KSHV initiated decay, the transcript il6 carries a 100 nucleotide long ARE in its 3′-UTR, which renders it resistant to SOX-mediated cleavage. Interestingly il6 mRNA is also resistant to HSV-1 VHS, suggesting that the host uses common escape mechanisms against host shut-off by different viruses. In the case of HSV-1 VHS RNase, both viral and cellular mRNAs are targeted if they are generated prior to infection. ARE-containing RNAs escape degradation after cleavage and are stored in P bodies [44]. Surprisingly some IRES- containing cellular RNAs not only escape decay but are rather induced by several fold (bip and apaf1 RNA) upon VHS expression [45]. 

In order to promote viral gene expression viral proteins need to distinguish between cellular and viral mRNAs, and avoid cellular RNA degradation machinery [46]. Many viruses lack the 5′-cap structure or the poly(A) tail, or both. Some viruses prevent 5′ to 3′ decay due to the complex secondary structure of internal ribosome entry sites (IRES) at their 5′ end in the absence of the cap structure. A small viral genome-linked protein (VPg) remains covalently attached to viral mRNAs in the absence of a protective 5′-cap for viral mRNAs of the *Picornaviridae* family [47], and facilitate translation of viral mRNAs of the *Caliciviridae* family [48]. In addition, hepatitis C virus (HCV) has a unique way of preventing decay of the viral mRNA by binding liver specific microRNA miR-122 [49,50]. Hyperpolyadenylation of cellular mRNAs upon KSHV infection may mark the RNA for degradation. Surprisingly, SCoV mRNA is both capped and polyadenylated, however, in an *in vitro*, assay it fails to get cleaved and degraded unlike cellular mRNAs [27]. Whether specific sequences in the viral RNA, or other viral proteins render SARS CoV mRNA resistant to RNA cleavage is yet to be investigated. HSV-1 VHS RNase selectively degrades stable cellular mRNAs and immediate early viral RNAs, but stabilizes host stress response mRNAs and viral late mRNAs by targeting AREs and the host protein tristetraprolin [44]. In summary, viruses employ one or multiple ways to down-regulate host gene expression at the level of mRNA while promoting stability of the viral RNA.

## 3. Shutting-Down Cellular Small Non-Coding RNAs

**Figure 4:**
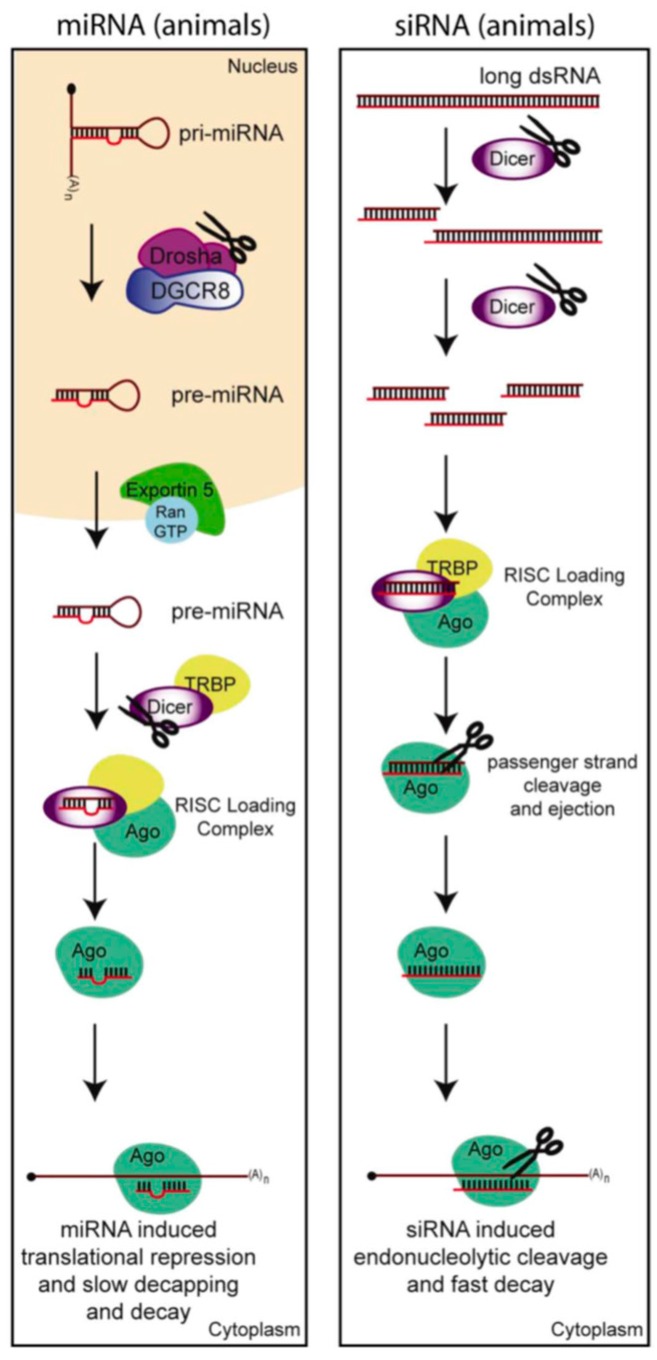
Major pathways for small RNA biogenesis and function. The major classes of RNAs involved in the endogenous RNAi response and that have been implicated in anti-viral function are microRNAs (miRNAs), and short-interfering RNAs (siRNAs), which are partially defined by their mechanisms of biogenesis and function. MiRNAs are typically derived from long Pol II transcripts, which fold into short imperfectly-basepaired hairpin structures. They undergo two cleavage steps by RNaseIII enzymes, Drosha in the nucleus and Dicer in the cytoplasm, to become ~22 nt dsRNAs. SiRNAs are normally derived from long perfectly-basepaired dsRNAs, which may result from convergent transcription, transcription of long inverted repeats, or by RNA dependent RNA Polymerases. Viruses often form dsRNA intermediates during their lifecycle. 22 bp products are produced by Dicer cleavage. SiRNAs are fairly uncommon in vertebrates; however, mammalian germ cells and embryonic stem cells appear to produce a variety of siRNAs from endogenous dsRNAs and long dsRNA [72,73,74]. One strand of the dsRNA is then incorporated into the RNA induced silencing complex (RISC) and becomes known as the miRNA or siRNA guide strand. A principal component of the RISC complex is an Argonaute (AGO) family protein which goes on to sequence-specifically recognize target mRNAs. MiRNAs typically show imperfect complementarity to their target sequences and therefore direct translational repression and a slow decapping and decay of the target mRNA. SiRNAs are perfectly complementary to their target mRNAs. This perfect complementarity activates the endonuclease or “slicer” activity of Ago so that the target is cleaved and quickly degraded.

The host shut-off effect is a phenomena used by viruses to silence the expression of host mRNAs, thereby diverting the cellular machinery to specifically translate viral messages. However, our view of RNA as a mere intermediate in the gene expression pathway has been challenged in recent decades by the discovery of many types of non-coding RNAs; here we will focus on the small non-coding RNAs involved in RNAi. RNAi pathways are a conserved eukaryotic mechanism, first discovered in fungi and plants [51,52], when gene silencing, rather than over-expression, was observed in response to foreign DNA being introduced. Work in *Caenorhabditis elegans* (*C. elegans*) later demonstrated that the mechanism of RNAi involved short (~22 nt) double-stranded RNA (dsRNA) induced silencing of complementary mRNAs [53]. The two primary classes of small RNAs that are involved in these silencing pathways are microRNAs (miRNAs) and short interfering RNAs (siRNAs) (Figure 4).

### 3.1. The Anti-Viral RNA Interference Pathway

In invertebrates, plants and fungi, the RNAi pathway represents the primary innate immunity anti-viral response. While siRNAs (Figure 4) may be derived from endogenous sources of dsRNA, all RNA viruses, except retroviruses, create long dsRNAs during their life cycle. Even DNA viruses might create long dsRNAs within structured RNA transcripts or by convergent transcription. These dsRNAs when processed by the cellular RNase III enzyme Dicer can yield siRNAs with perfect complementarity to the RNA genome of RNA viruses or viral transcripts of either RNA or DNA viruses, thereby destabilizing the genome or shutting down expression of viral transcripts. Thus, at least in plants, nematodes, arthropods, and fungi, which possess the ability to process long dsRNA into siRNAs, RNAi appears to be a critical antiviral intrinsic immune response [54,55,56]. In these cases where the RNAi pathway has a clear anti-viral function, viruses have launched a counter-defense in the form of viral suppressors of RNAi (VSRs) [57]. VSRs may silence the RNAi pathway through several mechanisms and some of the first and most varied examples of each of these mechanisms have been identified in plant viruses. They can either: (1) bind and inactivate any of the small RNA biogenesis machinery [58,59,60,61,62,63]; or (2) they can directly bind the RNA to prevent its processing into small RNAs, prevent loading into the RNA induced silencing complex (RISC), or induce its degradation [64,65,66,67,68,69]; or they can (3) prevent the initial expression of the small RNA biogenesis machinery or precursor dsRNAs [55,70,71]. While VSRs can in theory silence any or all of the small non-coding RNAs in the RNAi pathways, as there is a lot of overlap in their biogenesis pathways, VSRs are normally considered proteins that silence the virally derived siRNAs that are known to play a role in the host’s intrinsic immune response in lower eukaryotes.

### 3.2. Proposed Anti-Viral Role of Small RNAs in Mammals

DsRNA also plays an important role in activating anti-viral responses in vertebrates. In mammals these viral dsRNA products are recognized by several pattern recognition receptors (PRRs), such as RIG-I, MDA5, and TLR3. These receptors activate the interferon (IFN) response, which is a hallmark of vertebrates and comprises a series of cytokines that are released by the infected cell to activate antiviral responses in a paracrine manner. In vertebrates, intracellular dsRNA can also directly activate the RNA-dependent protein kinase (PKR) and 2'-5' oligoadenylate synthetase (OAS). The former phosphorylates the eukaryotic translation initiation factor 2α (eIF2α), leading to the inhibition of translation of both viral and cellular transcripts. The latter stimulates RNA cleavage by RNase L, leading to mRNA and ribosomal RNA (rRNA) degradation [75]. 

Whether RNAi is an important anti-viral system in vertebrate somatic cells, where the interferon response predominates, is debated [76]. Mammals themselves express very few endogenously encoded siRNAs but instead express mostly microRNAs (miRNAs) (Figure 4), since human Dicer processes pre-miRNA hairpins at a much higher rate than long dsRNA substrates [77]. SiRNAs of viral origin have been undetectable in most studies of infected mammalian somatic cells [78,79]. However, when the picornavirus encephalomyocarditis virus (EMCV) infects embryonic stem cells (ESCs), it produces readily detectable levels of virally derived siRNAs and differentiation of the cells dramatically decreases their ability to produce virally derived siRNAs. These data suggest that there is an inherent difference in the ability of ESCs and somatic cells to produce virally derived siRNAs [80]. This is particularly interesting as the IFN response is not active in oocytes and embryonic stem cells (ES cells) [81].

Since most of the enzymes are conserved between the biogenesis pathways of mammalian miRNAs and lower eukaryotic siRNAs it is tempting to speculate that eukaryotic miRNAs or the biogenesis machinery might also play an anti-viral role. The idea that the biogenesis machinery itself could have an anti-viral role, was in fact found for Drosha. Drosha relocates from the nucleus to the cytoplasm upon infection by various RNA viruses (SINV, VSV), where it can restrict the propagation of the virus by cleaving its RNA genome. This activity was independent of small RNA production or the IFN system [82]. The idea that small RNAs, like miRNAs, may play an anti-viral role in mammalian cells is supported by the fact that several labs have engineered viruses that can be targeted by miRNAs [83,84,85].

Another way to prove that the small RNA pathways are anti-viral is by showing that the virus expresses proteins to suppress small RNAs, such as the VSRs described above. VACV infection of mammalian cells has been shown to result in the polyadenylation and subsequent degradation of host miRNAs [86]. The larger subunit of the virally encoded heterodimeric poly(A) polymerase, VP55, was shown to be necessary and sufficient for this small RNA tailing and degradation either upon VACV infection or upon infection by a recombinant VSV virus that encodes VP55 [86,87]. Restoring miRNA function during VACV infection does inhibit virus replication, which could point to an anti-viral role for the host miRNAs and a miRNA suppressive role for VACV VP55 [86], but when recombinant VSV is created to express, VP55 viral titers are actually attenuated instead of enhanced, as the decrease in miRNAs leads to an enhancement of the Interferon response [87].

The data on VSRs in the arthropod-borne RNA viruses of the genus *Flavivirus*, such as dengue (DENV) and West Nile virus (WNV), is similarly non-conclusive. One investigation of DENV infection of mammalian Huh7 cells showed a general down-regulation of mammalian miRNAs and their biogenesis factors. They further showed that the DENV NS4B protein was able to suppress miRNAs, even when its domain that is known to suppress IFN signaling was deleted [88]. However, the suppression of the miRNA pathway in DENV infected cells is at odds with work showing that DENV-2 can be engineered to be targeted by miRNAs and that DENV-2 does not interfere with the accumulation of several endogenous miRNAs [83]. DENV and WNV use XRN1 to generate a noncoding structured subgenomic flavivirus RNA (sfRNA) that inhibits interferon signaling. The sfRNA is created by stalling of XRN1 at a pseudoknot structure within the 3′-UTR of the viral genomic RNA [89]. The stalling of XRN1 and presumably slow release from sfRNA, inhibits its enzymatic activity in the cell and leads to an increase in the half-life of host mRNAs [90]. Mammalian cells infected with mutant viruses, defective in sfRNA generation, show reduced virulence and pathogenicity. The accumulation of a structured RNA that would be expected to activate the immune system actually, instead, results in sustained anti-viral IFN signaling in mammalian cells [89,91]. The sfRNAs, of the flaviviruses, have also been shown to suppress both siRNAs and miRNAs through inhibiting Dicer function in both human and insect cells [92]. However, since the miRNAs suppressed by sfRNAs are not shown to be specifically anti-viral in mammals these are not generally considered VSRs. 

Some other proposed VSRs in mammalian viruses are the human immunodeficiency virus type 1 (HIV-1) Tat protein [93], Influenza virus NS1 protein, VACV E3L [94], Ebola virus VP35 [95], Nodamura virus B2, and primate foamy virus Tas [96]. Although, more recent studies do not find that HIV-1 Tat or influenza NS1 actually suppress RNAi [85,97]. One of the problems with demonstrating that these proteins indeed function as VSRs, is that their VSR function has been ascribed to their dsRBDs. By binding dsRNA, they can also block the IFN response. The specificity of these proteins as VSRs is called into question, by the fact that overexpression of even a bacterial dsRBD containing protein could function as a VSR in plants [98]. Additionally, an influenza virus lacking NS1, its putative VSR, was still lethal in interferon defective mice, suggesting either that the RNAi response is not sufficient to suppress the virus or that other VSRs are yet to be found [99]. 

To our knowledge, the only genetic rescue of VSR-deficient viruses in RNAi-deficient mammalian host cells was achieved for NoV replication in ESCs [80]. Given that these cells lack the IFN response, this represents convincing evidence for a VSR function of the NoV B2 protein and an anti-viral role for RNAi in ESCs. 

While it appears that vertebrate somatic cells might not generally use virally-derived siRNAs or endogenous miRNAs for anti-viral functions, interestingly, many vertebrate viruses have co-opted the RNAi machinery for pro-viral functions. Viral infection often alters host-cell miRNA expression to its advantage [100,101]. In addition, many viruses are known to encode miRNAs in their own genomes. In some cases, these miRNAs might even be homologs of the cellular miRNAs, as has been observed for KSHV which expresses a miR-155 homolog [102]. These are normally viruses with a nuclear DNA component to their life-cycle so that they can take advantage of the full complement of the host cells miRNA biogenesis machinery. The viruses that encode miRNAs also normally induce persistent latent infections, as expression of microRNAs rather than proteins might represent an advantage as they can go undetected by the host cell’s adaptive immune response. In these cases, viral miRNAs generally promote latent infections by either promoting cell longevity, evasion of the host cell immune response, or limiting lytic cycle reactivation [103]. 

### 3.3. Suppression of Selected miRNAs by Mammalian Viruses

The complete suppression of small RNAs in many viruses would be disadvantageous. In fact, as discussed above HCV actually requires the host liver specific miR-122 to stabilize and replicate its RNA genome [50] and similarly the replication, stability, and translation efficiency of the pestivirus genome is also dependent on interactions between the RNA genome with miR-17 and let-7 [104]. It was recently found that the HCV and pestivirus genomes, by “sponging” host miR-122 and miR-17 respectively, caused a derepression of specific target mRNAs and in the case of HCV this sponging created an environment favoring oncogenic potential [105]. Suppression of small RNA biogenesis would also be disadvantageous to viruses that encode their own miRNAs within their genome. These also happen to be viruses that normally establish long-term latent infections in their host. The viruses that have been firmly established to express their own miRNAs are the herpesviruses (only one of eight human herpesviruses (varicella zoster virus; VZV) does not encode its own miRNAs), adenoviruses, and polyomaviruses [79,103]. Many of these virally-encoded miRNAs or virally upregulated miRNAs have been shown to play a critical role in the viral life cycle. A complete shutdown of small RNAs in these viruses would therefore not be advantageous, but of course viral infection is a highly disruptive process that induces many gene expression changes. This includes significant changes in miRNA expression levels. While some of these miRNA levels may change in order to favor the viral life cycle, many may also change in order to defend the cell against viral infection.

**Figure 5:**
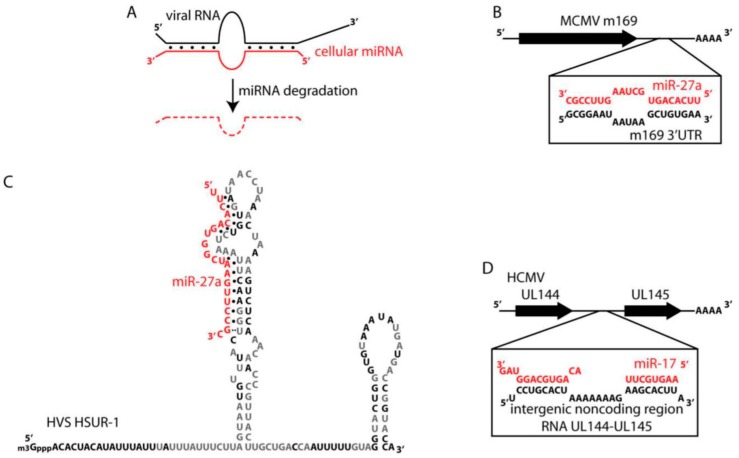
Viral RNAs can induce cellular miRNA degradation. (**A**) Generalized scheme showing a viral RNA (in black) bound to a cellular miRNA (in red). In at least three documented examples this type of binding induces miRNA degradation. (**B**) The 3’ UTR of mouse cytomegalovirus (mCMV) *m169* binds to and induces the degradation of miR-27 in host cells. (**C**) The noncoding RNA of herpesvirus saimiri (HVS) HSUR-1 also binds to and induces the degradation of miR-27. The sequence and predicted secondary structure of HSUR-1 are shown. Black nucleotides are perfectly conserved in all available genome sequences from independent isolates of HVS A, B, and C strains and also in *Herpesvirus ateles*. Predicted basepairs and non-canonical basepairs are by black dots or double dots, respectively. (**D**) The intergenic noncoding region in UL144-145 RNA of human cytomegalovirus (hCMV) binds and induces the degradation of miR-17.

Given our focus on ways in which viruses antagonize mammalian gene expression we would like to highlight studies that show viral-induced degradation of specific host miRNAs. Interestingly, three studies all found in different herpesviruses that virally expressed RNAs induce the degradation of specific host miRNAs (Figure 5A). This is contrary to the normal expectation that the miRNA induces the degradation of its RNA targets. In 2010, two studies reported that miR-27 was selectively degraded in response to infection by either mouse cytomegalovirus (mCMV) or herpesvirus saimiri (HVS) [106,107,108]. Later, it was found that five out of seven of the miR-17 family of miRNAs, miR-17, miR-20a, miR-20b, miR-93 and miR-106b, are degraded upon human cytomegalovirus (hCMV) infection [109]. In each case, the miRNAs are degraded in response to being bound by specific virally-expressed RNAs that bear no evolutionary relationship to one another [110]. In the case of mCMV, miR-27 is bound to the 3’ UTR of mCMV’s m169 gene (Figure 5B). Mutation of the binding site within the m169 gene resulted in virus attenuation upon *in vivo* infection [111]. In HVS, miR-27 is bound by a small non-coding RNA, HSUR-1, which resembles the snRNAs of eukaryotic cells (Figure 5C) and the authors show that the binding site in HSUR-1 can be mutated to target miR-20a for degradation instead [107]. The mRNA targets of miR-27 in T-cells were enriched for the T-cell receptor-signaling pathway, therefore HVS infection is expected to modulate T-cell activation and potentially transformation [112]. A non-coding region of an RNA was also responsible for binding and degradation of the miR-17 family of miRNAs in hCMV (Figure 5D). The function of this RNA was required for fast viral replication during the lytic cycle [109].

Little is known about the mechanisms for this RNA-induced degradation of miRNAs in herpesviruses. One possible mechanism could entail the recruitment of exonucleases to the miRNA by sequence elements within the target RNA. As HSUR-1 is itself degraded by an ARE-dependent pathway, it was hypothesized that the ARE within HSUR-1 might recruit exonucleases that also degrade miR-27. However, mutation of the ARE within HSUR-1 simply stabilized HSUR-1 and actually more significantly decreased the levels of miR-27. Therefore miR-27 appears to be degraded in an ARE-independent pathway [107]. So far the most likely model for RNA-induced degradation of miRNAs comes from a study showing that miRNAs with a high degree of complementarity at their 3’ ends to their targets, show increased non-templated nucleotide addition to their 3’ ends. This tailing was then associated with trimming and degradation of the miRNAs [113]. The proposed mechanism is that target complementarity to the 3’ end of the miRNA exposes the 3’ end of the miRNA to tailing enzymes, which then precedes degradation by exonucleases [110]. Consistent with this model, it was shown that miR-27 shows increased tailing and trimming in response to mCMV infection [111]. Despite these observations of miRNA-induced degradation by viral RNAs, little is still understood about how miRNAs get turned-over in general. Where in the cell does miRNA degradation happen? Which exonucleases are involved? If tailing is a prerequisite to degradation, which terminal transferases are required and is there a difference in function between 3’ end adenylation and uridylation? While these three examples illustrate RNA-induced degradation of specific miRNAs, do other viruses use protein-based mechanisms that might be more similar to the VSRs described in non-vertebrate viruses?

In summary, viruses manipulate their host cell’s gene expression patterns significantly to their advantage. In the case of lower eukaryotes, where the RNAi pathway is clearly anti-viral, the viruses have launched their own defense in the form of VSRs. In higher eukaryotic somatic cells, viruses generally focus on shutting down the interferon response. However, they have also co-opted the miRNA pathway to their own advantage in order to manipulate the cellular transcriptome. Some viruses utilize miRNAs to stabilize and replicate their genomes, thereby sponging the miRNAs away from their normal host targets [104]. Some express their own miRNAs, and finally others have developed clever strategies to down-regulate particular cellular miRNAs.

## 4. Conclusions

Viral infection, whether the virus is highly pathogenic or infects relatively benignly, either because it establishes a persistent non-pathogenic infection or establishes a latent infection until lytic reactivation, has significant effects on the host cell gene expression. While the cell attempts to detect and eliminate foreign invasive nucleic acids, the virus manipulates the cell’s gene expression pathways for many purposes. Many viruses attempt to hijack the cellular machinery to translate their own messages rather than cellular messages in an effort to maximize viral proliferation. Other viruses that establish persistent infections might rather focus on evading the cellular immune responses. In this review, we have discussed how viruses antagonize host mRNAs or small noncoding RNAs of the RNAi pathway to their advantage. Despite the compact genomes of most viruses several also genomically encode their own non-coding RNAs. Several of these noncoding RNAs were discussed in this article. In the future we will likely also find that viruses significantly affect the expression of cellular long noncoding RNAs. We still have much to learn about the interplay between viruses and their host-cell.

## Figures and Tables

**Table 1:** Viruses highlighted.

Classification of Virus (Genome Type)	Family	Virus	Characteristics
I: Double-stranded DNA (dsDNA)	Herpesviridae	Epstein Barr Virus (EBV)	Linear dsDNA genome remains encased in enveloped viral capsid Viral DNA is replicated and transcribed in the cell’s nucleus Life cycle has separate latent and infectious lytic phase
		Kaposi Sarcoma Herpes Virus (KSHV)	
		Herpes Simplex Virus 1 (HSV-1)	
		Herpes Virus Saimiri (HVS)	
		Cytomegalovirus (CMV)	
	Adenoviridae		Nonenveloped nuclear capsid encases dsDNA genome Nuclear replication Early and late phase life cycle
	Poxviridae	Vaccinia virus (VACV)	Enveloped with dsDNA genome Replicates exclusively in the cytoplasm
III: Double-stranded RNA (dsRNA)	Reoviridae	Rotavirus	Nonenveloped virion with linear dsRNA genome Replication is cytoplasmic
IV: Single-stranded (+) sense RNA ((+) ssRNA)	Coronaviridae	Human Coronavirus (HCoV)	Spiked, enveloped virion with nucleocapsid ssRNA is capped and polyadenylated Replication is cytoplasmic
		Severe Acute Respiratory Syndrome Corona Virus (SARS CoV)	
		Middle East respiratory syndrome corona virus (MERS CoV)	
	Picornaviridae	Poliovirus	Nonenveloped virion with linear ssRNA genome mRNA is protected by 5’ linkage to VpG protein, polyadenylated
		Encephalomyocarditis virus (EMCV)	
	Caliciviridae		Nonenveloped virion with linear RNA genome mRNA is protected by 5’ linkage to VpG protein, polyadenylated Replication is cytoplasmic
	Flaviviridae	Hepatitis C Virus (HCV)	Enveloped virion mRNA is without a poly(A) tail but can be capped (WNV and DENV) or uncapped (HCV) Replication is cytoplasmic
		Dengue Virus (DENV)	
		West Nile Virus (WNV)	
	Togaviridae	Sindbis Virus (SINV)	Enveloped virion mRNA is capped, polyadenylated Replication is cytoplasmic
	Nodaviridae	Nodamura Virus (NoV)	Non-enveloped virion with linear RNA genome mRNA is capped but not polyadenylated Replication is cytoplasmic
V: Single-stranded (-) sense RNA ((-) RNA)	Bunyaviridae	Rift Valley Fever Virus (RVFV)	Negative stranded enveloped virus with RNA genome mRNA is capped by cap snatching but is not polyadenylated Replication is cytoplasmic
	Orthomyxoviridae	Influenza Virus	Enveloped virion contains linear ssRNA genome mRNA is capped by cap snatching and polyadenylated Replication is nuclear
	Rhabdoviridae	Vesicular Somatic Virus (VSV)	Enveloped virus with linear ssRNA genome mRNA is capped and polyadenylated Replication is cytoplasmic
	Filoviridae	Ebola virus	Filamentous virus mRNA is capped and polyadenylated Replication is cytoplasmic
VI: Single-stranded (+) sense RNA with DNA intermediate in life-cycle	Retroviridae	HIV-1 (human immunodeficiency virus type 1)	Enveloped virion contains two ssRNAs that are capped and polyadenylated Replication is nuclear
		Primate foamy virus	

**Table 2:** Summary of mRNA suppression mechanisms.

Type of mRNA Suppression	Virus	Family	Viral protein and mechanism
mRNA Transcription	poliovirus	*Picornaviridae*	Protease 3C mediated cleavage of TFIID
	VSV	*Rhabdoviridae*	Protein M mediated inhibition of TFIID
	RVFV	*Bunyaviridae*	NSs mediated inhibition of TFIIH
	Influenza	*Orthomyxoviridae*	NS1 blocks RNA processing by CPSF, PAB, U6 RNA
Post-transcription suppression of mRNA	RVFV	*Bunyaviridae*	Nucleocapsid protein mediated 5′-cap removal
	Influenza	*Orthomyxoviridae*	PB2 mediated 5′-cap removal, PA mediated RNA cleavage
	Poxvirus SINV	*Poxviridae* *Togaviridae*	D9 and D10 mediated decapping of mRNA HuR capture by viral 3′UTR
Translational inhibition with and without mRNA cleavage	KSHV	*Herpesviridae*	SOX mediated mRNA cleavage
	EBV	*Herpesviridae*	BGLF5 mediated mRNA cleavage
	HSV	*Herpesviridae*	HSV-1 RNase mediated mRNA cleavage
	HCoV	*Coronaviridae*	NSP1 mediated ribosome stalling
	SARS CoV	*Coronaviridae*	NSP1 mediated ribosome stalling followed by cleavage by unknown cellular protein
	MERSCoV	*Coronaviridae*	NSP1 mediated ribosome stalling and cleavage by unknown nuclease
